# Nanomechanics of Multi-Walled Carbon Nanotubes Growth Coupled with Morphological Dynamics of Catalyst Particles

**DOI:** 10.3390/nano15181441

**Published:** 2025-09-19

**Authors:** Shuze Zhu

**Affiliations:** Department of Engineering Mechanics, Zhejiang University, Hangzhou 310000, China; shuzezhu@zju.edu.cn

**Keywords:** nanomechanical modeling, molecular dynamics simulations, carbon nanotube growth

## Abstract

Low-dimensional carbon nanostructures such as nanotubes, nanocones, and nanofibers can be grown in chemical vapor deposition (CVD) synthesis using catalyst nanoparticles. It is commonly observed that the morphology of solid catalyst nanoparticles continuously fluctuates during multi-walled carbon nanotube (MWCNT) growth. Interestingly, when the diameter of the inner tube of the growing MWCNT reduces below a threshold value, the catalyst nanoparticle snaps out of the MWCNT and recovers its spherical shape. If the MWCNT is tapered, the catalyst nanoparticle may also break. In this study, large-scale molecular dynamics simulations and nanomechanical modeling are employed to elucidate the complete process of MWCNT growth coupled with morphological change in the catalytic nanoparticles. It is shown that the tendency to decrease the surface energy of the catalyst nanoparticle is the major underlying driving force for the variation in morphology under the mechanical constraint of the growing MWCNT. Importantly, the predicted critical inner CNT radius at the onset of the shape recovery is in excellent agreement with experimental observations. The combination of molecular dynamics simulations and theoretical modeling offer an alternative perspective on co-evolution of catalyst nanoparticles and the growth of low-dimensional carbon nanostructures.

## 1. Introduction

Carbon nanotube (CNT) [1,2] is a characteristic low-dimensional nanostructure, which can be produced by chemical vapor deposition (CVD) [3,4], using catalyst nanoparticles such as Ni [5,6,7]. It has been found that the morphological evolution of the catalyst nanoparticles during CNT nucleation and growth will affect the resulting structures of CNT. The transmission electron microscope [8,9,10] reveals that the catalyst remains crystalline and solid during nucleation and growth [11,12]. It is commonly observed that the catalyst particles elongate, contract, and occasionally break during the growth of multi-walled carbon nanotube (MWCNT) structure [8,13]. Particularly [13], a portion of the Ni catalyst nanoparticle (about 5 nm in diameter) elongates and radially contracts inside the growing MWCNT. Interestingly, after the inner tube diameter decreases to about 2.5 nm, the particle recovers its original spherical shape by snapping out of the MWCNT confinement. The current study proposes a nanomechanics perspective to quantify such effect. Occasionally, because the innermost tube diameter becomes non-uniform and decreases along growth direction, the nanoparticle fractures into two parts, as complete snapping out is challenged by a much-increased energy barrier. Focusing on a 5 nm diameter Ni particle in the current study is largely motivated from a variety of experimental observations [13,14,15].

The investigation of the correlation between catalyst particle morphology and CNT growth is thus important for controllable synthesis. While computational effect and theoretical modeling have been reported to explain the reshaping behavior of large crystalline Ni catalyst particles [13,14,15,16,17,18], the complete process of MWCNT growth coupled with morphological change in the catalytic nanoparticles has not been studied with atomistic simulation details [19,20]. The complexity is that the continuing growth of more walls reduces the inner radius of the tubular structure where the nanoparticle resides, while the gradually elongated axial morphology determines whether snap-out or fracture happens in nanoparticles. In this study, large-scale molecular dynamics simulations and nanomechanical modeling are presented to elucidate experimental observations on the growth of MWCNT [13]. The input parameters for these simulations closely align with experimental settings. Based on simulation results, nanomechanical analysis on the morphological dynamics of catalyst nanoparticles is provided, which reproduces the experiment-agreeable critical diameter to induce snap-out process. The current work offers alternative insights to interpret the catalytic growth of low-dimensional carbon nanostructures.

## 2. Simulation Methods

Large-scale atomic/molecular massively parallel simulator (LAMMPS) [21] is used to perform molecular dynamics simulations. Each CNT wall is modeled as rigid bodies. The non-bonded interaction between carbon atoms in the CNT (except those at the tube rim) and the Ni atoms in the catalyst nanoparticle is modeled by the Lennard-Jones 12-6 potential with the parameters ϵ_Ni-C_ = 0.00172 eV, σ_Ni-C_ = 0.2978 nm as developed through the customary Lorentz-Berthelot mixing rules, using Ni-Ni and C-C parameters directly extracted from the Universal Force Field [22]. The interaction between carbon atoms with dangling bonds at the tube rim and the Ni atoms is modeled with a set of enhanced parameters ϵ_Ni-C_ = 0.1 eV, σ_Ni-C_ = 0.2 nm to simulate the anchoring effect at the step edge. Such convenient treatment on the anchoring effect (the rim has a much stronger interaction strength) is based on various literature interpretations on experimental observation [13,15]. Embedded atom (EAM) potential file with the LAMMPS distribution is used to describe the metallic bond interaction between Ni atoms in the catalyst nanoparticle. The EAM potential has been developed for many types of metals. For example, in the case of Ni, the sublimation energy is 4.45 eV and the vacancy-formation energy is 1.63 eV [23]. The simulation is performed on a canonical ensemble. The kinetic energy of the Ni nanoparticle is controlled by a Nosé–Hoover thermostat at 1500 K. The newly added CNT wall first constricts radially, starting from the final configuration of the previous CNT until there is a 0.34 nm radial distance from the previous CNT wall, and then elongates for a certain amount of time until another new inner wall is added. During the addition of walls every 50 ps, all the dynamics quantities such as the temperature and velocity of the nanoparticle atoms are inherited. Loop coding is utilized in LAMMPS script to enable a continuous step-wise process for adding walls in a single simulation run. The CNT growth velocity is 0.02719 nm/ps. Using the experimental CNT growth velocity is computationally prohibitive. The outmost CNT diameter is 5.64 nm. Periodic boundary condition is not imposed along the axial growth direction. Each simulation is repeated five times to ensure that the results are convergent and statistically sound. The influences from nanoparticle size, mechanical properties of flexible CNT walls [24], chirality of CNT walls, structural defects from CNT walls, and temperature gradient over larger space [25] require future investigations. 

## 3. Results and Discussions

**Figure 1:**
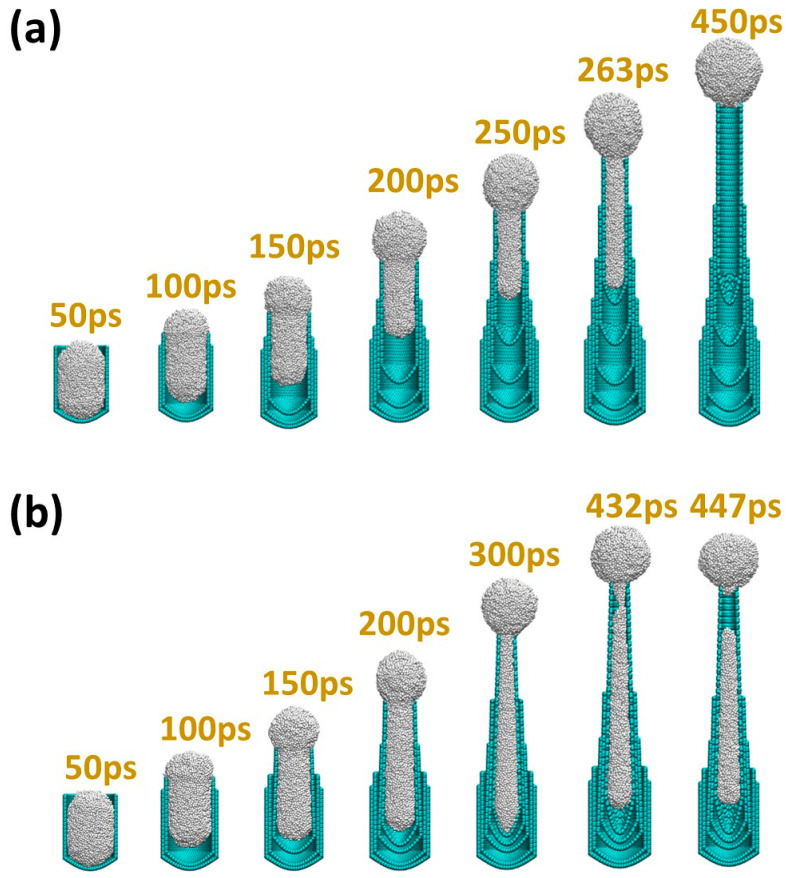
Molecular dynamics simulations of the morphological evolution of the catalyst nanoparticle during MWCNT growth. (**a**) The particle snaps out of the end of the MWCNT and recovers to a spherical shape when the CNT walls grow straight. (**b**) The particle cannot completely snap out due to a tapered inner CNT wall and eventually fractures into two parts.

A Ni particle with 5 nm diameter was initially placed in a section of a 5.64 nm diameter CNT with a carbon cap and its shape elongated inside the CNT (Figure 1a, at 50 ps). The interaction between carbon atoms in the CNT (except those at the tube rim) and the Ni atoms in the particle was modeled as non-bonded. For the carbon atoms at the tube rim, a stronger interaction with the surface Ni atoms was applied to simulate the CNT rim anchoring at the surface steps as observed experimentally. The detachment of the particle from the CNT cap occurred when the particle tended to recover its shape and moved out of the tube. Meanwhile, a smaller diameter inner tube with a conical end (cap) was inserted around the particle to simulate the inner tube formation. The inserted inner tubes always contain caps, which is consistent with the experimental observations [13], and are always longer than the outer ones, which is due to the difference in growth rate of the inner layer and outer layer. Once the inner tube was inserted, the outer layer detached from the Ni particle, and its growth terminated.

The inner layer remained at a distance of 0.34 nm from the neighboring outer layer; thereby, the inner tube diameter reduced to 4.98 nm (Figure 1a, at 100 ps). The smaller diameter tube caused the particle diameter to decrease. The particle length increased while keeping its volume constant, and its tendency for shape recovery results in a particle detachment from the inner cap. After the particle detached from the inner cap, a smaller inner tube was inserted again (Figure 1a, at 150 ps). During the cycles of particle detachment and inner tube insertion, the particle elongated more and more as its diameter continued to reduce to keep up with the diameter of the inner tube (Figure 1a, from 200 ps to 263 ps). After inserting the sixth layer tube with a diameter of 2.28 nm, the particle completely snapped out of the tube and recovered into a round shape, which agrees well with the experimental observations [13] (Figure 1).

Figure 1b shows another simulation that begins with a 5 nm Ni nanoparticle in a 5.64 nm CNT with a cap (Figure 1b, at 50 ps). However, upon the nucleation of the third inner tube, a tube with a tapered end—the axial radius of the rim region is 0.34 nm smaller than that of the cap region—was inserted (Figure 1b, at 150 ps). The diameter of the particle that was in contact with the inner tube also decreased from the cap end to the tube rim region. The tendency of particle shape recovery was similar to inner tubes with uniform diameters. However, further growth of the inner tube led to the breaking of the particle into two parts at the tube rim region: an elongated part left inside the tube and another round part snapped out of the tube, which is also consistent with the experimental observations [13].

**Figure 2:**
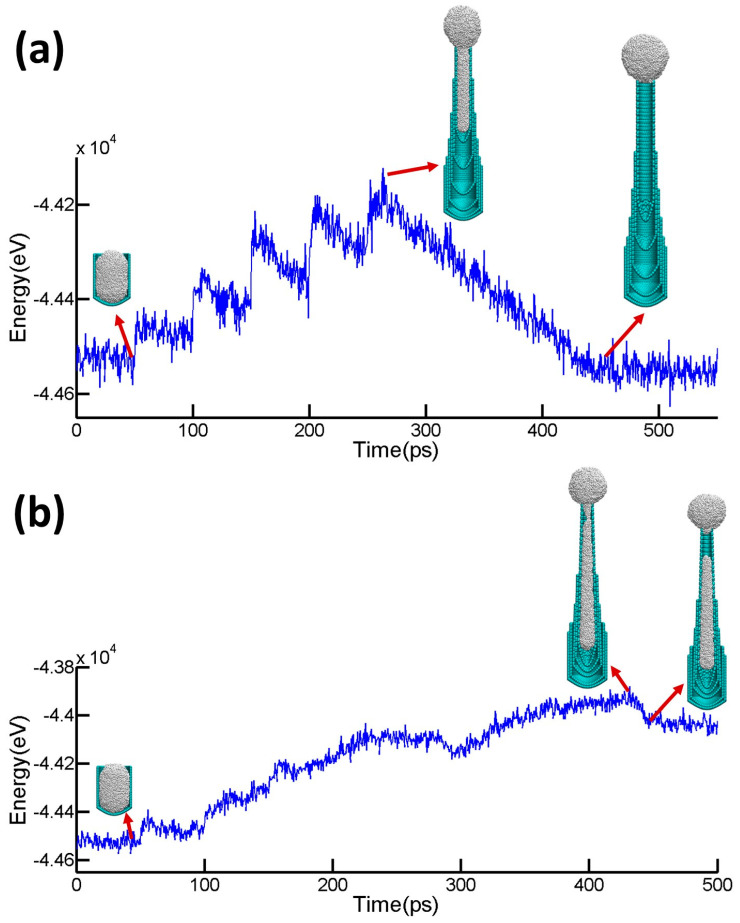
The evolution of the total potential energy for the nanoparticle during MWCNT growth with straight walls (**a**) and tapered inner walls (**b**).

The above molecular dynamics simulations can capture the two representative modes of the morphological evolution of the catalyst nanoparticles during MWCNT growth well and can be further explained by the following energetic understanding. Figure 2a plots the total potential energy of the simulation model (i.e., combination of the surface energy of the catalyst nanoparticle and the adhesion energy between the nanoparticle and the MWCNT) corresponding to Figure 1a as a function of simulation time. Figure 2 clearly shows that the nucleation of each new inner layer inside the existing layers of the tube leads to a sharp increase in the total potential energy of the nanoparticle, which can be attributed to the associated increase in strain energy of the catalyst nanoparticle due to the mechanical constraint of the new inner tube and the increase in the surface area (thus surface energy) of the catalyst nanoparticle due to particle elongation. As the newly nucleated inner tube grows, the total potential energy gradually decreases. Such a decrease is mainly due to the shape change in the nanoparticle (shortening) at the end of the new inner tube (the tube rim) and part of the nanoparticle moving out of the CNT to form a hemisphere, both of which relax the strain energy and reduce the surface energy of the nanoparticle. Eventually, when the whole nanoparticle snaps out of the CNT and adapts into a round shape, the total potential energy further decreases and finally reaches a level comparable to that of the original state of the catalyst nanoparticle. Previous studies [15] have shown that the reshaping of the particle is driven by a capillary-driven surface diffusion process that features a fast collective diffusion on the catalyst inside the tube and reduced Ni mobility on steps where CNTs are attached, in agreement with the present simulations.

Figure 2b plots the total potential energy of the model corresponding to Figure 1b as a function of simulation time. Similar sharp increases in total potential energy upon the nucleation of each new inner layer of the tube are apparent. After the nucleation and growth of a tapered inner layer, the stress level in the catalyst nanoparticle near the rim continues to increase. The shape of the tapered inner tube also increases the energy barrier for the atoms in the catalyst nanoparticle to move toward the thinner end of the inner tube (see Figure S1 in Supplementary Information for details). As a result, the growth of the tapered inner tube forces the further elongation of the portion of the catalyst nanoparticle that remains inside the CNT, which causes a continuous increase in surface area and stress level of the nanoparticle. The increasing tensile stress in the nanoparticle eventually fractures the nanoparticle into two parts, with one part snapping out of the tube and morphing into a round shape, and another part still elongated inside the tube. Consequently, the total potential energy stabilizes to a higher level than that of the original state of the catalyst nanoparticle.

**Figure 3:**
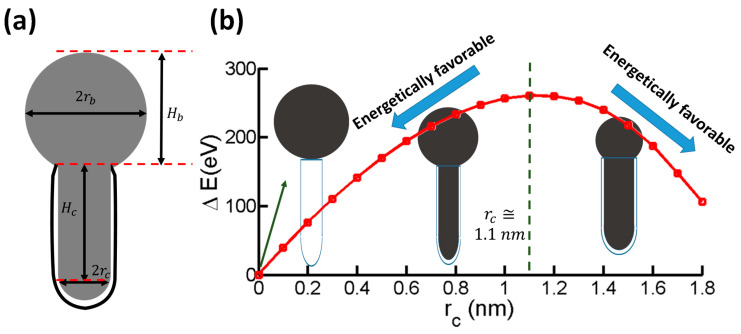
(**a**) Model of a typical configuration of nanoparticle, which consists of a partial sphere, a cylinder, and a half sphere. (**b**) Variation in the total free energy as a function of $r_{c}$ when $H_{c}$ is fixed to be 5.3 nm, comparable to the nanoparticle as in experiment.

To further offer mechanistic understanding of the shape recovery of the nanoparticle, the nanomechanical energetic interplay during the morphing process is considered (see Figure S2 in Supplementary Information for details). Figure 3a depicts a catalyst nanoparticle in the shape as shown in Figure 1. The total volume of the nanoparticle (about 73.36 nm^3^) is calculated from

(1) $$V_{0}=\pi r_{c}^{2}H_{c}+\frac{\pi H_{b}\left(3r_{c}^{2}+H_{b}^{2}\right)}{6}+\frac{2}{3}\pi r_{c}^{3}$$

The total free energy of the system is given by

(2) $$E_{total}=E_{adh}+E_{surf}$$

In Equation (1), the adhesion energy $E_{adh}$ between CNT and nanoparticle is integrated numerically (see Figure S2 in Supplementary Information for details). The total surface energy of the nanoparticle (as shown in Figure 3a) $E_{surf}$ is calculated by

(3) $$E_{surf}=\gamma (2\pi r_{c}H_{c}+2\pi r_{b}H_{b}+2\pi r_{c}^{2})$$

where γ is taken [26] as 2 J/m^2^.

At each event of new carbon inner tube initiation and growth with a smaller radius, the portion of the nanoparticle inside the CNT is subject to a radial compression, which is expected to build up an increment of strain energy. However, given the high mobility of the atoms in the nanoparticle at high temperature during the CNT growth, the portion of the nanoparticle inside the CNT can quickly rearrange its atomic configuration to effectively relax the increased strain energy.

Figure 3b plots the variation in the total free energy as a function of $r_{c}$ when $H_{c}$ is fixed to be 5.3 nm, comparable to the nanoparticle as in Figure 1. There exists a peak value of the total free energy at $r_{c}=$ 1.1 nm. In other words, when the radius of the inner CNT is greater than 1.1 nm, the nanoparticle prefers to stay inside the inner CNT. As the new inner CNT layer nucleates and grows, the cylindrical portion of the nanoparticle is forced to decrease. When the radius of the inner CNT becomes smaller than 1.1 nm, it becomes more energetically favorable for the cylindrical portion of the nanoparticle to morph out of the inner CNT and recover its spherical shape outside the CNT, as suggested by the decreasing trend of the total free energy in Figure 3b. The above prediction of the shape recovery process of the nanoparticle and the critical inner CNT radius ($r_{c}=$ 1.1 nm) at the onset of the shape recovery is in excellent agreement with experiments [13]. The decreasing trend of the total free energy as $r_{c}$ gets smaller becomes even steeper, suggesting that the smaller the inner CNT radius, the faster the shape recovery rate. Further analysis (see Supplementary Information) shows that the surface energy takes up most of the system energy. For example, it becomes two orders of magnitude higher than the adhesion energy, e.g., at the critical value of $r_{c}=1.1\;nm$, $E_{surf}=1333.27\;eV$, $E_{adh}=-13.20\;eV$. Such a comparison suggests that surface energy is the dominating parameter that governs the shape-morphing of the nanoparticle during CNT growth. Further theoretical analysis also shows that the threshold value is robust over slightly enlarged interfacial adhesion energy (see Figure S3). Also note that the current model assumption on straight walls is not suitable for quantitative investigating of the catalyst fracture process, as the experimental process of particle fracture is much more complicated in the sense that the subsequent grown CNT walls will seal the nanoparticle inside, leading to a more stabilized nanoparticle encased by the CNT walls. Future extensions on models are needed to account for catalyst fracture.

## 4. Conclusions

Molecular dynamics simulations of morphological behavior of catalyst nanoparticles during MWCNT growth are performed, capturing the experimental phenomena where the catalyst particles elongate, snap out, and occasionally break during growth. It is found that the increase in potential energy due to the mechanical deformation and surface energy of the catalyst nanoparticle is the underlying driving force for the variation in catalyst nanoparticle morphology under the mechanical constraint of the growing MWCNT. The present analysis of the competing energetics suggests that the surface energy is the dominating parameter in driving the shape recovery of the catalyst nanoparticle. Importantly, the predicted critical inner CNT radius at the onset of the shape recovery is in excellent agreement with experimental observations. The current work provides alternative elucidation on the mechanism governing the co-evolution of catalyst nanoparticles and the growth of low-dimensional carbon nanostructures.

## Data Availability

Data is contained within the article or Supplementary Material.

## Supplementary Materials

The following supporting information can be downloaded at: https://www.mdpi.com/article/10.3390/nano15181441/s1, Figure S1: Variation of the total potential energy of a small-sized nanoparticle moves (1141 Ni atoms ) in a conical shape CNT as same as the final configuration of CNT walls in Figure 1b.The nanoparticle tends to move toward the bottom and morphs its shape with an increasing diameter. The potential energy decreases as the particle moves and morphs. Figure S2. (a) Model of a typical configuration of nanoparticle, which consists of a cylinder (labeled as part (1)) and a semisphere end (part (2)) and a partial sphere head (part (5)), inside the inner CNT (cylindrical part (3) and semispherical cap part (4)). (b,c): top view and side view of the parts (1), (2), (3), (4) in (a). Table S1: Calculation of data points in Figure 3b. Figure S3. The dependence of the adhesion energy per area. The figure shows that the larger the adhesion energy per area, the smaller the theoretical critical radius. Up to 1 eV/ nm^2^, the prediction matches well on the experimental result. For a variety of adhesion energy per area, we do see an energy barrier, which corresponds to the theoretical critical radius. More simulation results and nanomechanical modeling details.
