# Supplementary material for: Nanomechanics of Multi-Walled Carbon Nanotubes Growth Coupled with Morphological Dynamics of Catalyst Particles
Source: Nanomaterials (Basel). 2025 Sep 19;15(18):1441. doi: 10.3390/nano15181441 (PMC12472867; doi:10.3390/nano15181441)
Supplement: Supplementary file 1 [file nanomaterials-15-01441-s001.zip › nanomaterials-3825705-supplementary.pdf]

Supplementary Information

Nanomechanics of multi-walled carbon nanotubes growth coupled with morphological dynamics of catalyst particles

Shuze Zhu[1*]

[1] Department of Engineering Mechanics, Zhejiang University, Hangzhou 310000, China
*To whom correspondence should be addressed. E-mail: shuzezhu@zju.edu.cn

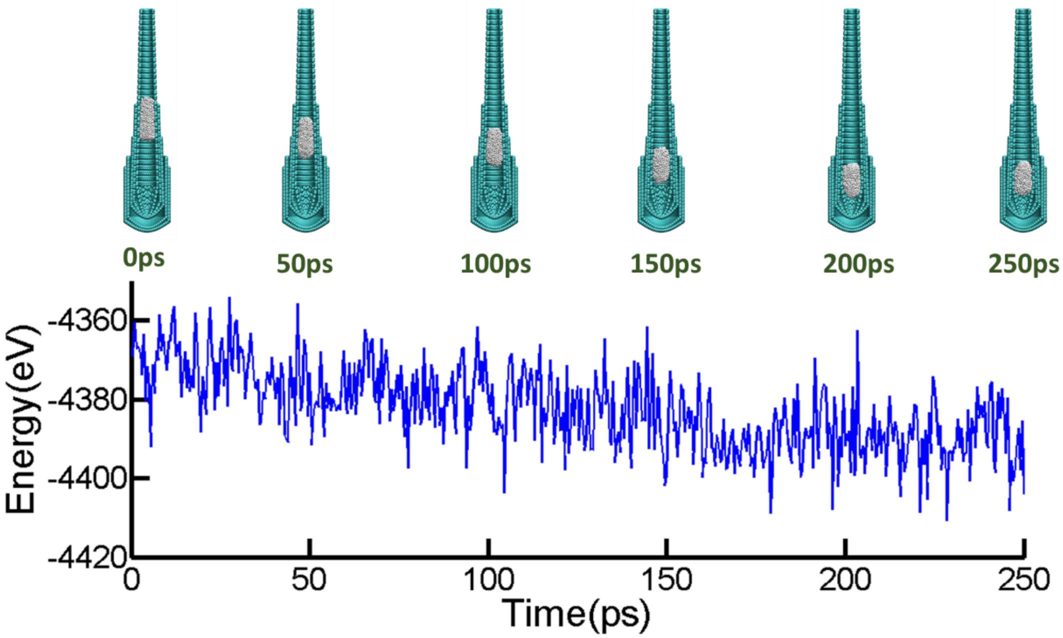

Figure S1: Variation of the total potential energy of a small-sized nanoparticle moves (1141 Ni atoms) in a conical shape CNT as same as the final configuration of CNT walls in Figure 1b.The nanoparticle tends to move toward the bottom and morphs its shape with an increasing diameter. The potential energy decreases as the particle moves and morphs.

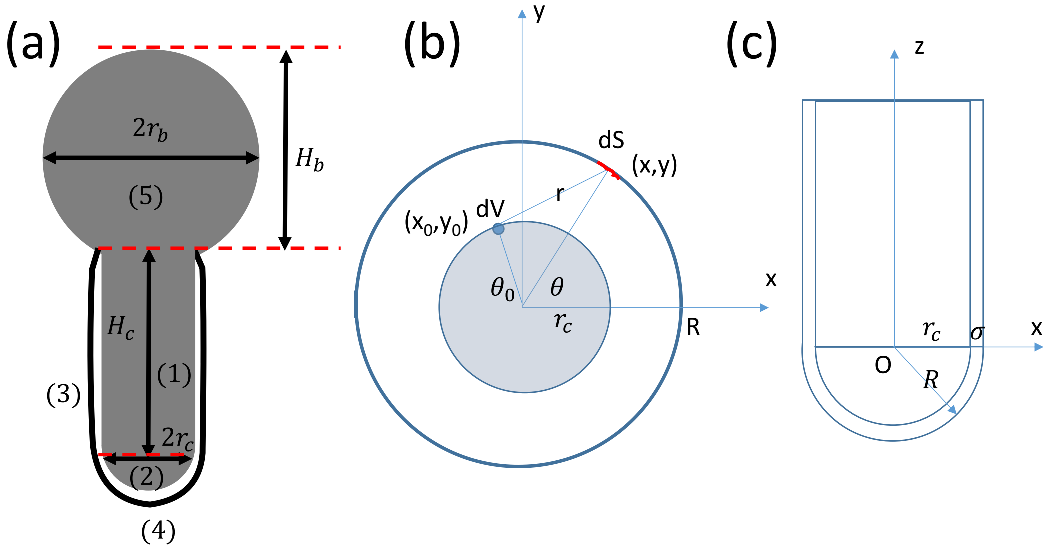

Figure S2. (a) Model of a typical configuration of nanoparticle, which consists of a cylinder (labeled as part (1)) and a semisphere end (part (2)) and a partial sphere head (part (5)), inside the inner CNT (cylindrical part (3) and semispherical cap part (4)). (b) and (c): top view and side view of the parts (1), (2), (3), (4) in (a).

Calculation of adhesion energy between the nanoparticle and the inner CNT:

Adhesion energy between the catalyst nanoparticle and the CNT consists of four parts:

$$\mathrm{E}_{adh} = E_{adh}^{(1-3)} + E_{adh}^{(2-4)} + E_{adh}^{(2-3)} + E_{adh}^{(1-4)}, \qquad \text{(S1)}$$

Including:

$E_{adh}^{(1-3)}$, the interaction energy between volume part (1) in the nanoparticle and surface part (3) in the CNT;

$E_{adh}^{(2-4)}$, the interaction energy between volume part (2) in the nanoparticle and surface part (4) in the CNT;

$E_{adh}^{(2-3)}$, the interaction energy between volume part (2) in the nanoparticle and surface part (3) in the CNT;

$E_{adh}^{(1-4)}$, the interaction energy between volume part (1) in the nanoparticle and surface part (4) in the CNT.

Given the decaying nature of the interaction between the nanoparticle atoms and the carbon atoms in the CNT and the large distance from majority portion of part (5) in the nanoparticle to the CNT, the interaction between part (5) and part (2) and that between part (5) and part (3) are expected to be negligible when compared with the above four terms in Eq. (S1), thus are not considered.

Interaction energy between volume part (1) in the nanoparticle and surface part (3) in CNT:

The coordinates of a material point in the nanoparticle are:

$$x_0 = r_0 cos\theta_0;\ y_0 = r_0 sin\theta_0;\ z_0 = z_0 \tag{S2}$$

The coordinates of a material point on the CNT surface are:

$$x = Rcos\theta; y = Rsin\theta; z = z \tag{S3}$$

The distance between these two material points is:

$$r^2 = (r_0 cos\theta_0 - Rcos\theta)^2 + (r_0 sin\theta_0 - Rsin\theta)^2 + (z_0 - z)^2 \tag{S4}$$

The adhesion energy between these two material points is modeled by vdW potential:

$$V(r) = 4\epsilon(\frac{\sigma^{12}}{r^{12}} - \frac{\sigma^6}{r^6}) \tag{S5}$$

The volume of an infinitesimal volume element in the nanoparticle is:

$$dV = r_0 dr_0 d\theta_0 dz_0 \tag{S6}$$

The area of an infinitesimal area element on CNT surface is:

$$dS = Rd\theta dz \tag{S7}$$

Therefore, the total interaction between dV of the nanoparticle and dS of the CNT is

$$\mathrm{d}E_{adh}^{(1-3)} = \rho R d\theta dz * \rho_0 r_0 dr_0 d\theta_0 dz_0 * V(r) \tag{S8}$$

ρ is atom density per area on the CNT surface, ρ_0 is the atom density per volume inside the particle.

Then one gets

$$E_{adh}^{(1-3)} = \int_0^{H_c} \int_0^{H_c} \int_0^{r_c} \int_0^{2\pi} \int_0^{2\pi} \mathrm{d}E_{adh}^{(1-3)}$$

$$=4\epsilon\rho R\rho_0 \int_0^{H_c}\int_0^{H_c}\int_0^{r_c}\int_0^{2\pi}\int_0^{2\pi} r_0\Big(\frac{\sigma^{12}}{\left(R^2+r_0^2-2Rr_0\cos(\theta-\theta_0)+(z-z_0)^2\right)^6}-$$

$$\frac{\sigma^6}{\left(R^2+r_0^2-2Rr_0\cos(\theta-\theta_0)+(z-z_0)^2\right)^3}\Big)d\theta d\theta_0\, dr_0 dz dz_0 \qquad \text{(S9)}$$

In a similar fashion, determine the other three parts as following.

Interaction energy between volume part (2) in the nanoparticle and surface part (4) in CNT:

The coordinates of a material point in the nanoparticle are:

$$x_0 = r_0 cos\theta_0 sin\phi_0; y_0 = r_0 sin\theta_0 sin\phi_0; z_0 = r_0 cos\phi_0 \qquad \text{(S10)}$$

The volume of an infinitesimal volume element in the nanoparticle is:

$$dV = r_0^2 sin\phi_0 dr_0 d\theta_0 d\phi_0 \qquad \text{(S11)}$$

The coordinates of a material point on the CNT surface are:

$$x = Rcos\theta sin\phi; y = Rsin\theta sin\phi; z = Rcos\phi \qquad \text{(S12)}$$

The area of an infinitesimal area element on the CNT surface is:

$$dS = R^2 sin\phi d\theta d\phi \qquad \text{(S13)}$$

ρ is atom density per area on the CNT surface, ρ_0 is the atom density per volume inside the particle.

$$E_{adh}^{(2-4)} =$$

$$4\epsilon\rho R^2\rho_0 \int_0^{r_c}\int_{\pi/2}^{\pi}\int_{\pi/2}^{\pi}\int_0^{2\pi}\int_0^{2\pi} r_0^2 sin\phi sin\phi_0 \left(\frac{\sigma^{12}}{((r_0cos\theta_0sin\phi_0-Rcos\theta sin\phi)^2+(r_0sin\theta_0sin\phi_0-Rsin\theta sin\phi)^2+(r_0cos\phi_0-Rcos\phi)^2)^6}-\right.$$

$$\left.\frac{\sigma^6}{((r_0cos\theta_0sin\phi_0-Rcos\theta sin\phi)^2+(r_0sin\theta_0sin\phi_0-Rsin\theta sin\phi)^2+(r_0cos\phi_0-Rcos\phi)^2)^3}\right) d\theta d\theta_0 d\phi d\phi_0 dr_0 \qquad \text{(S14)}$$

Interaction energy between volume part (2) in the nanoparticle and surface part (3) in CNT:

The coordinates of a material point in the nanoparticle are:

$$x_0 = r_0 cos\theta_0 sin\phi_0; y_0 = r_0 sin\theta_0 sin\phi_0; z_0 = r_0 cos\phi_0 \qquad \text{(S15)}$$

The volume of an infinitesimal volume element in the nanoparticle is:

$$dV = r_0^2 sin\phi_0 dr_0 d\theta_0 d\phi_0 \quad \text{(S16)}$$

The coordinates of a material point on the CNT surface are:

$$x = Rcos\theta; y = Rsin\theta; z = z \quad \text{(S17)}$$

The area of an infinitesimal area element on the CNT surface is:

$$dS = Rd\theta dz \quad \text{(S18)}$$

ρ is atom density per area on the CNT surface, ρ_0 is the atom density per volume inside the particle.

$$E_{adh}^{(2-3)} = 4\epsilon\rho R\rho_0 \int_0^{H_c}\int_0^{r_c}\int_{\pi/2}^{\pi}\int_0^{2\pi}\int_0^{2\pi} r_0^2 sin\phi_0 \left(\frac{\sigma^{12}}{((r_0cos\theta_0sin\phi_0-Rcos\theta)^2+(r_0sin\theta_0sin\phi_0-Rsin\theta)^2+(r_0cos\phi_0-z)^2)^6} - \frac{\sigma^6}{((r_0cos\theta_0sin\phi_0-Rcos\theta)^2+(r_0sin\theta_0sin\phi_0-Rsin\theta)^2+(r_0cos\phi_0-z)^2)^3}\right) d\theta d\theta_0 d\phi_0 dr_0\, dz \quad \text{(S19)}$$

Interaction energy between volume part (1) in the nanoparticle and surface part (4) in CNT:

The coordinates of a material point in the nanoparticle are:

$$x_0 = r_0cos\theta_0; y_0 = r_0sin\theta_0; z_0 = z_0 \quad \text{(S20)}$$

The volume of an infinitesimal volume element in the nanoparticle is:

$$dV = r_0dr_0d\theta_0dz_0 \quad \text{(S21)}$$

The coordinates of a material point on the CNT surface are:

$$x = Rcos\theta sin\phi; y = Rsin\theta sin\phi; z = Rcos\phi \quad \text{(S22)}$$

The area of an infinitesimal area element on the CNT surface is:

$$dS = R^2 sin\phi d\theta d\phi \quad \text{(S23)}$$

ρ is atom density per area on the CNT surface, ρ_0 is the atom density per volume inside the particle.

$$E_{adh}^{(1-4)} = 4\epsilon\rho R^2\rho_0 \int_0^{H_c}\int_0^{r_c}\int_{\pi/2}^{\pi}\int_0^{2\pi}\int_0^{2\pi} r_0 sin\phi \left(\frac{\sigma^{12}}{((r_0cos\theta_0 - Rcos\theta sin\phi)^2+(r_0sin\theta_0 - Rsin\theta sin\phi)^2+(z_0-Rcos\phi)^2)^6} - \frac{\sigma^6}{((r_0cos\theta_0 - Rcos\theta sin\phi)^2+(r_0sin\theta_0 - Rsin\theta sin\phi)^2+(z_0-Rcos\phi)^2)^3}\right) d\theta d\theta_0 d\phi dr_0\, dz_0 \quad \text{(S24)}$$

The following parameters are used in calculation of adhesion energy: $\epsilon = 0.00172\ eV$, $\sigma = 0.2978\ nm$, $\rho = 38.1781\ nm^{-2}$, and $\rho_0 = 91.646\ nm^{-3}$.

Table S1: Calculation of data points in Fig. 3(b)

H_c (nm)	r_c(nm)	E_{surf}(eV)	E_{adh}(eV)	E_{total}(eV)	dE(eV)
0	0	1059.3	0	1059.3	0
5.3	0.1	1099.69	-0.77384	1098.916	39.61616
5.3	0.2	1137.54	-1.94855	1135.591	76.29145
5.3	0.3	1172.7	-3.12365	1169.576	110.2764
5.3	0.4	1205.01	-4.3305	1200.68	141.3795
5.3	0.5	1234.31	-5.46872	1228.841	169.5413
5.3	0.6	1260.38	-6.67412	1253.706	194.4059
5.3	0.7	1283.02	-7.92062	1275.099	215.7994
5.3	0.8	1301.96	-9.20942	1292.751	233.4506
5.3	0.9	1316.9	-10.502	1306.398	247.098
5.3	1	1327.48	-11.8349	1315.645	256.3451
5.3	1.1	1333.27	-13.2046	1320.065	260.7654
5.3	1.2	1333.76	-14.7085	1319.052	259.7515
5.3	1.3	1328.3	-15.8306	1312.469	253.1694
5.3	1.4	1316.1	-17.2547	1298.845	239.5453
5.3	1.5	1296.15	-18.9929	1277.157	217.8571
5.3	1.6	1267.36	-20.5116	1246.848	187.5484
5.3	1.7	1229.39	-22.1414	1207.249	147.9486
5.3	1.8	1189.86	-24.1328	1165.727	106.4272

Effect of perturbed interfacial adhesion energy

In the calculation, after obtaining the total adhesion energy E_{adh}, dividing this value by the total contact area between NP and CNT leads to the adhesion energy per area. For the set of parameters in the main text, this adhesion energy per area is calculated to be ~0.3 eV/nm^2, corresponding to $\epsilon = 0.00172\, eV$. Then by perturbing such ϵ value, the adhesion energy per area is proportionally changed. A set of results is shown in Figure S3, where up to 1 eV/ nm^2, the prediction matches well on the experimental result.

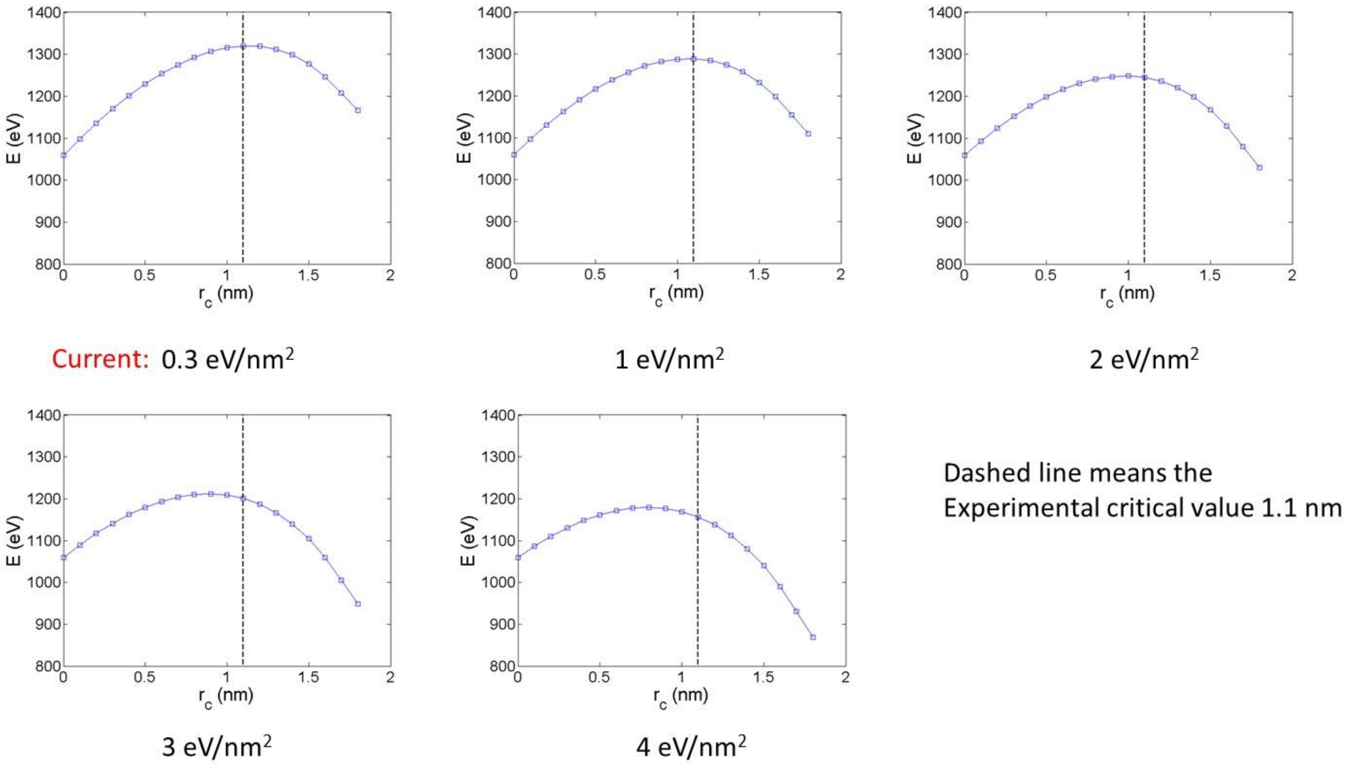

Figure S3. The dependence of the adhesion energy per area. The figure shows that the larger the adhesion energy per area, the smaller the theoretical critical radius. Up to 1 eV/ nm^2, the prediction matches well on the experimental result. For a variety of adhesion energy per area, we do see an energy barrier, which corresponds to the theoretical critical radius.
